# Human Temperaments. XIII.—The Suspicious Temperament


**Published:** 1916-10-21

**Authors:** 


### HUMAN TEMPERAMENTS.

### XIII.—The Suspicious Temperament.*

The suspicious temperament shows itself to the observing eye in early life, but nevertheless it is often overlooked at that stage, and even when fully developed in early manhood receives insufficient attention. It is important, however, that it should be recognised, for it is apt to develop into one of the most intractable and dangerous forms of madness.

The child of suspicious temperament is different from other children. He is not given to sociable and boisterous play, but prefers to sit or ramble by himself, indulging in reveries and castle-building. He lives in an imaginary world of romance, in which he is, of course, the central and heroic figure. He is studious, and usually has a fair share of ability, so that he takes a good place in his class, and is well thought of by his masters, but he is not popular with his schoolmates. He keeps aloof from them, and they reciprocate by sending him to Coventry, not formally and designedly, but practically. Schoolboys are as a rule cruel little brutes. They are cruel, not of set purpose, but from want of sympathy. As the embryo starts as a unicellular organism, and passes through the various stages of development that the race has passed through in its progress from the unicellular organism to man, so the child after birth passes through the various stages of human development that are traversed by man in his progress from the state of savagery to that of high civilisation.

### The Psychology of the Schoolboy.

The young schoolboy is a savage, and as he grows older gradually emerges from savagery into barbarism. In both stages he is cruel, and in both stages he shares with the savage and the barbarian their detestation and contempt of the stranger and the independent, non-conforming person who departs from the wonted and accepted ways of customary and common life. The schoolboy who refuses to conform to schoolboy usages, but strikes out an independent line of his own, is not popular with other schoolboys, and is made to feel his unpopularity not only in being sent to Coventry, but also by more positive action, which is the sweetest sorrow to the boy of suspicious temperament. He glories in feeling that he is under-estimated, misunderstood, unappreciated, and misjudged. He does not complain, but he hugs his misery, indulges in orgies of self-pity, retires more and more into himself, and accentuates his singularity.

When he leaves school and goes out into the world, he is already a morbid being. He is self-centred, and his interests and attention are not externalised, but turned inward upon himself. More and more he becomes peculiar and different from other men, and if we can penetrate into his mind, we find at the bottom of his peculiarity a colossal conceit. He is in his own opinion the cleverest, the ablest, the best, the handsomest, the most deserving of men. And yet he cannot disguise from himself that his successes and achievements are by no means proportionate to his merits. For this he has an easy and manifest explanation. He is still undervalued, misjudged, misunderstood, under-estimated. His ill-success is due to no deficiency of his own, but to the ignorance, jealousy, favouritism, and malice of other people. The defect is not in his own achievements, which are splendid enough, but in the stupidity or the malice of others, which cannot recognise, or refuses to admit his superiority. He does not complain loudly, but he adds this new grievance to the reckoning, and scores another injustice against mankind.

### The Slings and Arrows of Outrageous Fortune.

He is subject to the little annoyances and troubles that beset us all, but he takes them much more seriously than other people, and attributes them to different and more sinister causes. Does he mislay a thing? He is sure it has been stolen. But it is of so little value that it is not worth stealing? Then it has been stolen out of malice, and for the purpose of annoyance. Does an acquaintance pass him in the street without recognising him? He is sure he has been intentionally cut. He has long suspected that acquaintance of unfriendly feelings towards him, and now his suspicions are confirmed. Does a tradesman make a mistake in executing an order for him? Such carelessness would not be allowed towards anyone else. Other people can be properly served. Other people's orders are executed with care, but he is considered of no account. It does not matter how he is served. Anything is good enough for him. Is one of the breakfast eggs bad? It is sure to fall to his share, and he will never believe that it fell so by accident. It was arranged that he should have it, or at least it came to him by some gross negligence that would have been avoided in the case of anyone else. And so he goes through life, always ill-treated, always selected and singled out for ill-treatment; always the victim of a carelessness and negligence that would never have been exercised towards anyone else; or worse, of malice, and of malice resting upon envy of his manifest superiority. But he does not complain—much. He prefers to hug his grievances, to pose as a martyr, to accept his slights and injuries with an ostentatious resignation that is more theatrical than Christian; or if he does display resentment, his resentment takes the form of sulks rather than of active anger. But if the fire does not blaze up brightly, it is none

* The previous articles appeared on May 6, 13, 27, June 10 and 24, July 8 and 22, Aug. 5 and 19, Sept. 9 and 23, and Oct. 7, 1916.

the less intense. It glows and smoulders in sullen internal fury, which may at length break out in raging flame, upon a provocation that is perhaps trivial in comparison with others that have been passed over.

If it goes no farther than this, the suspicious temperament may be regarded as still restricted within the bounds of the normal; but it is not always thus restricted, and at best it is the normal representative or degree of a mental peculiarity that, when exaggerated so as to pass beyond the bounds of the normal, becomes one of the most striking, the most peculiar, the most dangerous, and the most incurable kinds of insanity. From imagining a constant succession of slights, injuries, injustices, and neglects to imagining a common cause for them all is not a long step. It is indeed almost a logical step. So many repeated experiences of the same kind are hardly consistent with fortuitous occurrence. If they really were, as the man of suspicious temperament believes, intentionally directed against him, it would look very much as if they were directed on a common plan from a common source, and gradually this conviction forces itself upon the suspicious man. He sees in every new occurrence of the kind, and almost everything that happens to him is to him an occurrence of the kind, fresh evidence of the existence of a plot against him. If we are much associated with him, we may witness the gradual conversion of the suspicion into a rooted conviction.

### The Boundary of Sanity.

Little by little we see a conjecture grow into a suspicion, the suspicion become more and more confirmed until it ripens into complete certainty. Then the delusion of persecution is established. Then the boundary of sanity is passed, and then whatever of reason there may have been in the suspicions, which was never very much, is scattered and abandoned. The morbid growth in the mind still continues, and there are now no bounds to its extravagance. Formerly some real annoyance, however slight, was needed in order to arouse the resentment. Now annoyance, and wilful, intentional annoyance, is attached to the most harmless and innocent occurrences, to occurrences that have no connection at all with the suspecting man. In every word, in every gesture and attitude of other people, he sees intention to injure him, or evidence of the plot to injure him. The casual greetings of strangers in the street have some reference to him and to the plot against him. The school children shouting at their play, and regardless of his presence, are jeering at him. Even the articles in the newspapers, while purporting to criticise the Prime Minister or the Home Secretary, really refer to him, and are using these personages as cover to attack him. The whole world is pressed into the service of the plot, and is utilised by his enemies to injure him. What astonishes us in all this is the colossal conceit that underlies it all. According to the diseased imagination of the paranoiac, he is the centre of universal attention. If his imaginings were true, the thoughts, the interests, the efforts and endeavours of the whole parish, the whole country, the whole world are concentrated upon him and absorbed in him. In his eyes the rest of creation is as interested in him as he himself is, and devotes to him all its time and attention. This also is carried to its logical conclusion. If he is so important that everyone is thinking of him and interested in him he must be some great personage; and accordingly he soon begins to imagine, first that he must be, and then that he is, a great personage. It is true that he occupies a very insignificant position, but this is because he is kept out of his rights by the machinations of his enemies. It is probable, and it soon becomes certain, that he was changed at birth. He is not the son of a City clerk or a country parson as he has always appeared to be. He is really of ducal, nay, of royal birth. He is the rightful heir to the throne. He should be by rights the actual occupant of the throne. He may go further than this and arrogate to himself the attributes of the Deity. There is no limit to his arrogance or to his assumptions. Such is the culmination of the suspicious temperament if its vagaries are allowed to proceed unchecked; and it is very difficult to put any curb upon them. The foundation of the temperament is conceit, and the most effectual way to take the conceit out of a man is to secure that he shall have plenty of traffic with the world, and shall be brought into contact with many men, each of whom, we may be sure, will excel him in some respect or other; but with the man of suspicious temperament this is difficult, for he is unsociable and solitary in taste and habit. The only method that offers much hope is to catch him young, to recognise and combat the peculiarity while he is still a child.

